# Multi class photoplethysmography-based deep model for cardiovascular disease classification

**DOI:** 10.1371/journal.pone.0347840

**Published:** 2026-05-15

**Authors:** Amjed Al Fahoum

**Affiliations:** Biomedical Systems and Informatics Engineering Department, Hijjawi Faculty for Engineering Technology, Yarmouk University, Irbid, Jordan; China Academy of Chinese Medical Sciences Institute of Chinese Materia Medica, CHINA

## Abstract

**Background:**

cardiovascular disease is the leading global cause of mortality. Photoplethysmography (PPG), widely embedded in consumer wearables, offers a scalable diagnostic modality. However, prior approaches are often constrained by handcrafted features, binary classification, and poor generalizability, limiting their clinical impact.

**Methods:**

A deep hierarchical convolutional neural network (CNN) was designed to extract both morphological and rhythmic characteristics directly from raw photoplethysmography (PPG) signals. The architecture employs progressively structured convolutional filter hierarchies to capture multi-scale signal features. To enhance signal stability and training efficiency, a dual-stage normalization strategy was implemented, consisting of Z-score standardization followed by Min–Max scaling. In addition, batch normalization and dropout regularization were incorporated to improve model generalization and reduce the risk of overfitting. The proposed framework was trained and evaluated on a multi-source dataset comprising 612 patients and 2,448 annotated PPG segments distributed across six diagnostic classes: atrial fibrillation (AF), heart failure (HF), acute coronary syndrome (ACS), cerebral vascular accident (CVA), deep vein thrombosis (DVT), and normal sinus rhythm (NSR).

**Results:**

The model achieved an overall accuracy of 93.48%, a macro-average F1-score of 0.9386, and a Cohen’s Kappa of 0.8968, indicating “almost perfect” agreement. AF and HF were detected with flawless precision and recall (1.000), while ACS achieved high sensitivity (recall 0.964). Errors were primarily confined to physiologically related conditions (e.g., ACS vs. CVA). Inference efficiency was demonstrated with <5 ms per segment on consumer-grade hardware, confirming feasibility for real-time applications.

**Conclusion:**

The proposed framework advances beyond lightweight but underpowered or overly complex models by combining representational depth with computational efficiency. Limitations include the need for external, multi-center validation and explainability integration. This study establishes a robust foundation for PPG-based, multi-class cardiovascular diagnostics, supporting clinical decision support and next-generation wearable health technologies.

## 1. Introduction

### 1.1. Background

Cardiovascular diseases (CVDs) remain the leading cause of mortality worldwide, responsible for nearly 18 million deaths each year [1]. Early detection and continuous monitoring are essential to enable timely intervention, improve outcomes, and ease the burden on healthcare systems. While electrocardiography (ECG) is the clinical gold standard for cardiac monitoring, its multi-electrode configuration and the need for clinical supervision limit its suitability for pervasive, long-term use [2]. Photoplethysmography (PPG), on the other hand, is a non-invasive, low-cost optical method that measures changes in blood volume at the microvascular level. PPG is a prevalent method for conducting extensive, decentralized health assessments and preventative cardiac care because of its extensive application in smartwatches and activity bands [3]. PPG serves as a valuable tool for context, quality assurance, and assessing cardio-metabolic risk. PPG has transitioned from simple pulse oximetry to a versatile platform for disease screening and cuffless hemodynamic monitoring. Early spectral methods such as MUSIC-based analysis on PPG demonstrated discriminative capacity between healthy and athletic subjects and established practical preprocessing and peak-detection considerations [4]. More recently, feature-selection pipelines on PPG have identified coronary artery disease with clinically relevant accuracy, emphasizing interpretable features and pragmatic selection strategies that can be repurposed for quality-aware decision support in home monitoring [5]. Deep-learning approaches using PPG scalograms and PPG-NET have shown encouraging results for non-invasive hypertension monitoring and for blood-pressure stage classification with pre-trained models [6–7]. Complementary engineering studies comparing piezoelectric, photosensor, and earlobe capture clarify trade-offs in signal fidelity and robustness across form factors—evidence directly applicable to selecting a PPG sensor site that coexists with a breath-sampling mouthpiece [8].

### 1.2. Problem Statement

The PPG waveform encodes a wealth of physiological information about cardiac pulsation, vascular elasticity, and autonomic control [9]. Alterations in waveform morphology, rhythm, or amplitude can reveal diverse pathologies ranging from arrhythmias to heart failure. Prior studies have demonstrated the use of PPG in detecting specific conditions such as atrial fibrillation (AF) [10] and sleep-related disorders [11]. However, a clear gap persists in developing robust, unified models capable of discriminating across multiple cardiac anomalies from raw PPG signals. This is primarily due to subtle inter-class morphological variations that traditional signal processing or shallow machine learning models struggle to capture [12].

### 1.3. Justification of the Problem

Existing approaches for multi-class CVD detection often depend on labor-intensive, hand-crafted feature extraction—such as crest time, reflection index, or stiffness indices—followed by conventional classifiers like Support Vector Machines or Random Forests [5,13]. While such methods can identify coarse distinctions, they are limited in capturing fine temporal details. Meanwhile, recent deep learning models optimized for wearables emphasize computational efficiency, but at times sacrifice the representational depth needed to characterize complex cardiac conditions [14,15].

### 1.4. Bridging the Gap: Proposed Approach

A deeper hierarchical 1D Convolutional Neural Network (CNN), trained end-to-end on raw PPG signals, is hypothesized in this work that it can capture the multiscale feature representations needed to distinguish multiple cardiovascular conditions with high fidelity. Unlike lightweight architectures that compromise expressive power, our proposed design consists of four stacked convolutional blocks of increasing depth. This design enables the progressive extraction of features, including high-level pathological characteristics and low-level pulse dynamics, without the need for manual feature engineering.

### 1.5. Main Highlights and Contributions

This research presents many contributions; among the main are the following:

1. **Innovative Architecture:** A specialized deep 1D-CNN engineered to exploit the temporal and morphological complexity of PPG signals for effective multi-class differentiation.
2. **End-to-End Learning:** The model obviates the need for manual feature engineering by learning directly from preprocessed raw PPG signals.
3. **Comprehensive Classification**: Demonstrates strong performance in distinguishing six cardiac conditions, extending beyond the narrow binary focus common in prior work.
4. **Robust Performance**: Achieves state-of-the-art accuracy (93.48%), confirming the importance of deeper architecture for complex PPG-based diagnostics.

### 1.6. Structure of the Article

The remainder of this paper is organized as follows. Section 2 reviews related work on PPG-based cardiovascular disease detection. Section 3 describes the dataset, preprocessing pipeline, and the proposed CNN model. Section 4 presents experimental results and comparisons. Section 5 discusses methodology, clinical implications, and interpretability. Section 6 sheds light on main limitations and future work. Section 7 concludes with a summary of findings.

## 2. Literature review

Photoplethysmography (PPG) has progressed from a companion to pulse oximetry into a clinically salient, information-dense signal for cardiovascular disease (CVD) diagnostics. Foundational work established the optical–hemodynamic basis—variations in transmitted/reflected light driven by pulsatile blood volume, vascular compliance, and wave reflections—and clarified how canonical landmarks (rapid systolic upstroke, dicrotic notch, diastolic decay) and their derivative representations encode vascular state [9]. Building on this physiology, derivative-domain analyses highlighted that second-derivative morphology tracks arterial aging and hypertension-related remodeling, anchoring a pathophysiology-first approach to PPG interpretation [16]. Collectively, these insights position PPG as a low-cost, scalable complement to electrocardiography for risk stratification, screening, and longitudinal follow-up across hypertension and arrhythmia detection.

Two methodological streams now co-define the field. The first leverages interpretable, handcrafted indices linked to vascular mechanics—timing intervals (e.g., upstroke/crest time, reflection indices), amplitude ratios, and derivative peaks—while emphasizing acquisition controls that materially affect cuffless blood pressure estimation (notably, sensor–skin contact pressure) [17]. The second embraces data-driven learning on raw waveforms or spectro-temporal encodings. Large-scale studies demonstrate that convolutional models outperform classical pipelines for heart-rate estimation under motion, establishing CNNs as robust feature extractors for ambulatory PPG [15]. For rhythm screening, PPG-based AF detectors have matured from rule-based to deep learning systems, with reviews synthesizing algorithmic performance and deployment considerations [18]; recent attention-based, multi-scale fusion strategies developed for unobtrusive cardio-mechanical signals underscore how multi-resolution temporal cues can be harnessed for arrhythmia detection in realistic conditions [19]. Generative pipelines further target artifact-restored wrist PPG to recover AF-relevant signatures in the wild, improving tolerance to motion and illumination shifts [20].

A coherent translational arc is visible in the PPG portfolio by Al-Fahoum and collaborators. Early work used spectral subspace (MUSIC) analysis to reveal phenotype-level distinctions in healthy versus athletic chores, demonstrating that PPG spectra carry discriminative cardiovascular information when appropriately modeled [4]. Subsequent clinical studies showed that a pragmatic, physiology-aware feature-selection pipeline on fingertip PPG can identify coronary artery disease, underscoring the utility of interpretable markers for supervised diagnosis [5]. In parallel, light-sensitive, scalogram-to-network pipelines (“PPG-NET”) achieved non-invasive hypertension monitoring, while pre-trained deep backbones paired with wavelet transforms supported blood-pressure stage classification, reinforcing the value of time–frequency representations for hemodynamic state decoding [6–7]. Importantly, comparative device studies (piezoelectric vs photosensor vs earlobe sites) mapped acquisition trade-offs that govern signal fidelity and robustness, offering practical guidance for clinical and consumer form factors [8].

The diagnostic horizon continues to broaden in two directions relevant to cardiovascular care. First, **quality-aware, edge PPG biometrics** using organic photodiodes (OPDs) and real-time quality checks (power spectral density, DC-drift screening, and deep learning) demonstrate that identity-specific morphology can be captured on IoT-grade hardware—an enabler for **personalized baselines** and trustable longitudinal thresholds in home monitoring [21]. Consistent with this, independent evaluations of PPG-based biometric systems benchmark deep architectures and pipelines for identity verification, strengthening the case that individualized cardiovascular phenotypes can be stably tracked across sessions and sensors [21–22]. Second, **sleep disordered breathing**, a major cardiovascular comorbidity, is now detectable from single-channel PPG with modern deep learning, opening a path to integrated cardiometabolic risk surveillance without adding new sensors [23]. Meanwhile, clinical epidemiology continues to emphasize **arterial stiffness** as a central risk construct; recent cohort data in former elderly smokers link stiffness with aging and smoking burden [24], motivating the use of PPG-derived surrogates (e.g., derivative indices and timing ratios) to screen for vascular remodeling alongside traditional measures.

Real-world evidence for PPG in cardiology is consolidating. Multicenter or randomized evaluations of smartphone/PPG AF screening indicate clinical traction and feasibility at population scale [25], while broader CVD-oriented pipelines apply machine learning to PPG for early risk detection and triage [26–27]. Throughout, diagnostic pathways remain anchored to clinical standards—e.g., the Fourth Universal Definition of Myocardial Infarction—clarifying where PPG augments ECG-centric workflows (pre-screening, monitoring, escalation triggers) rather than replacing confirmatory testing [28].

Two persistent challenges shape current agendas: artifact robustness and generalization. Motion, ambient light fluctuations, contact pressure, and skin–sensor coupling degrade PPG quality; comparative work shows that adaptive filtering and deep denoisers can materially improve downstream detection, provided validation avoids overfitting to sensor- or motion-specific signatures [29]. At scale, domain shift across demographics, devices, sampling sites, and collection protocols degrades out-of-distribution performance; comprehensive reviews underline the need for subject-wise validation, domain adaptation, and stratified reporting by hardware and skin tone [30]. For blood-pressure estimation, a recent systematic review and meta-analysis highlights variability in study quality and performance metrics, reinforcing the need for standardized protocols and external testing [31]. Open datasets—such as smartwatch AF corpora—are catalyzing reproducible benchmarks and ablation practices, though multicenter, device-diverse datasets tied to clinical end points remain a priority [32].

Deployment requirements are steering innovation toward **efficient** and **explainable** models. Real-time, on-device HRV and rhythm analytics with PPG on embedded platforms demonstrate that clinically useful inference is feasible within the computational budgets of wearables, tightening the loop between algorithm design and edge implementation [33]. In parallel, integrated explainability—e.g., saliency over scalograms or structured waveform matrices—improves transparency into which morphological regions drive classification, aiding clinician trust, post-market surveillance, and failure analysis [34]. Within this trajectory, Al-Fahoum’s body of work provides a methodologically grounded scaffold—from interpretable feature pipelines and disease-focused deep architectures to acquisition-centric engineering—that supports the next generation of clinically targeted, scalable PPG diagnostics.

## 3. Materials and methods

### 3.1. Data acquisition and preprocessing pipeline

**Fig 1:**
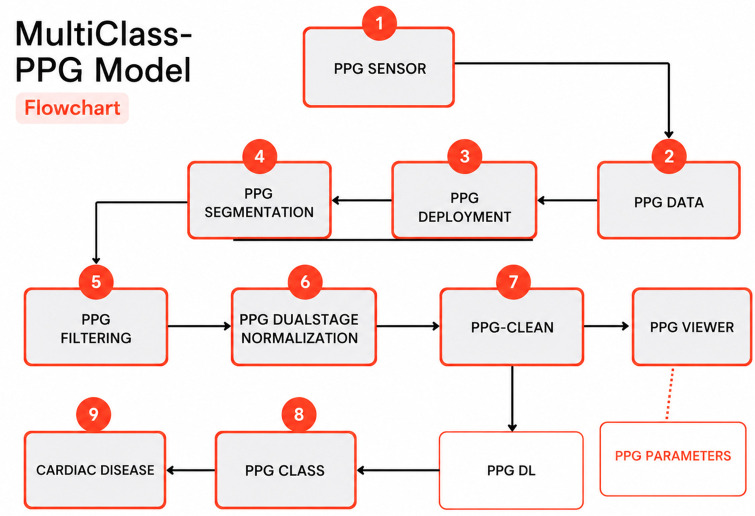
Block diagram of the multiclass-PPG model.

High-quality, consistently processed PPG is essential for reliable learning. The dataset comprised 2,448 labeled PPG segments pooled from public corpora (e.g., clinical PPG-BP derivatives, MIMIC-style sources) and curated clinical collections, with cardiologist-confirmed labels for AF, HF, ACS, CVA, DVT, and Normal. Fixed 12-second windows (~12–20 beats at 60–100 bpm) were used to balance temporal context with stationarity, enabling simultaneous capture of rhythm irregularity (AF) and morphology-linked pathology (HF) without excessive label drift—an approach consistent with reviews of PPG deep learning and AF screening in real-world smartphone workflows [18,30,35–38]. Fig 1 shows the block diagram of the multiclass-PPG model.

#### 3.1.1. Multi-stage denoising and standardization.

PPG susceptibility to motion, contact pressure, ambient light, and device variation was addressed via a staged pipeline informed by established physiology and recent signal-quality research. A zero-phase 64th-order FIR bandpass (0.5–12.5 Hz) suppressed baseline wander and high-frequency noise while preserving notch/diastolic content and reflective wave morphology critical for vascular and BP surrogates [9,13,36,39]. The passband upper edge (≈12.5 Hz) retains fine-scale slope changes and notch dynamics (linked to dicrotic notch physics and reflection timing), supporting downstream notch/crest timing and derivative-domain analyses [13,40].

**Dual-stage normalization followed:** per-segment Z-score (mean 0, variance 1) to mitigate amplitude dispersion from perfusion, site, or coupling; then min–max to [0,1] for bounded inputs and stable gradients. Normalization harmonizes distributions across devices and subjects—an essential step highlighted by deep-learning reviews and real-world validations to reduce domain shift and improve calibration [30,35,38,41].

#### 3.1.2. Signal quality and artifact controls.

Prior to segmentation and model ingestion, signal-quality indexing (SQI) and artifact screens were applied: (i) PSD-based checks for cardio-dominant bands and (ii) DC-drift thresholds, aligned with emerging SQI best practices for remote PPG and OPD-based IoT implementations [42–43]. To further harden against motion, a lightweight artifact detector (trained on augmented PPG with synthetic motion/illumination perturbations) flagged or excluded segments, following recent edge-oriented motion-artifact detection strategies (e.g., Tiny-PPG) [15,44]. This stack yields standardized, physiologically faithful inputs and directly addresses the generalization issues often reported in cross-device PPG studies [30,37,38,41].

### 3.2. Dataset composition and clinical characteristics

The dataset analyzed in this study comprises 2,448 photoplethysmography (PPG) signal segments derived from 612 unique patients collected from a multi-source clinical pool. Data was obtained from a tertiary-care cardiovascular center and affiliated diagnostic laboratories between 2016 and 2024 under institutional approval. All diagnoses were confirmed by board-certified cardiologists based on clinical examination, imaging, laboratory findings, and established diagnostic criteria.

The distribution of unique patients across diagnostic classes was as follows:

- Atrial Fibrillation (AF): 94 patients
- Heart Failure (HF): 88 patients
- Acute Coronary Syndrome (ACS): 102 patients
- Cerebrovascular Accident (CVA): 96 patients
- Deep Vein Thrombosis (DVT): 87 patients
- Normal Sinus Rhythm (NSR): 145 patients

The mean age of the cohort was 57.4 ± 13.2 years, with 58% male and 42% female participants. Comorbidities such as hypertension and diabetes were recorded when available but were not used as predictive features.

Inclusion criteria consisted of:

1. Confirmed clinical diagnosis of one of the target conditions.
2. High-quality PPG recording of at least 12 seconds duration.
3. Age ≥ 18 years.

Exclusion criteria included:

1. Severe motion artifacts compromise signal morphology.
2. Incomplete clinical documentation.
3. Coexisting acute systemic instability.

The study protocol was approved by the Institutional Review Board of the participating healthcare institutions using the IRB/2016/215, IRB/2021/115, and IRB/2024/333, and all data were anonymized prior to analysis.

### 3.3. Patient-wise splitting and data leakage control

#### 3.3.1. Data partitioning strategy and leakage prevention.

To ensure valid generalization assessment, all data splitting was performed at the patient level rather than the segment level. Each individual contributed data exclusively to one subset (training, validation, or testing). No segments from the same patient were distributed across multiple splits.

The dataset was partitioned as follows:

- Training: 80% of patients
- Validation: 10% of patients
- Test: 10% of patients

Stratification was applied at the patient level to preserve class distribution balance across subsets.

This subject-wise separation prevents information leakage arising from repeated morphological patterns of the same individual appearing in both training and testing sets. Consequently, reported performance reflects generalization to unseen patients rather than memorization of subject-specific signatures.

### 3.4. Proposed deep hierarchical convolutional neural network architecture

Robust **multi-class** PPG diagnosis benefits from **hierarchically structured** models that learn multi-scale temporal morphology—not solely ultra-lightweight AF-specific designs. The proposed 1D-CNN comprises **four convolutional blocks** (filters 32 → 64 → 128 → 256; kernels 13–15) with **BN-ReLU-MaxPool** in each block, followed by **global average pooling** and a **regularized classifier head** (dense-128 with L2 = 0.01, dropout = 0.5; softmax). The table you provided already enumerates roles and clinical advantages; below are literature-anchored design rationales.

#### 3.4.1. Architecture overview.

The network comprises four sequential convolutional blocks with increasing filter depth, followed by a classification head. Table 1 summarizes the hierarchical CNN architecture for PPG analysis, detailing each layer’s specifications, functional role, and clinical advantages, highlighting how progressive feature extraction enhances robustness, interpretability, and multi-class cardiovascular classification accuracy. This hierarchy enables progressive learning from basic pulse structures to pathological morphologies:

**Table 1:** Structured Representation of the Deep CNN for PPG Signal Analysis.

Stage	Layer (Name)	Specifications	Functional Role	Advantages for PPG Signal
Input	Sequence Input (input)	1D input channel, MinLength = *minLen*	Accepts preprocessed PPG segments	Preserves temporal order of raw PPG waveform
Block 1 – Low-Level Feature Detector	Conv1D (conv1)	Kernel = 13, Filters = 32, Padding = same	Extracts basic edges & local fluctuations	Captures beat-to-beat variations & fine-scale noise patterns
	Batch Norm (bn1)	Normalization	Stabilizes learning, reduces internal covariate shift	Handles amplitude variability due to sensor or skin tone differences
	ReLU (relu1)	Non-linearity	Introduces feature selectivity	Detects subtle local peaks and troughs
	MaxPooling1D (pool1)	Pool size = 2, Stride = 2	Downsamples while retaining key features	Reduces sensitivity to noise and artifacts
Block 2 – Mid-Level Pattern Integrator	Conv1D (conv2)	Kernel = 15, Filters = 64	Extracts broader temporal patterns	Learns short-term rhythm structures across pulses
	Batch Norm (bn2), ReLU (relu2), MaxPool (pool2)	Same principles as above	Stabilizes and compresses features	Enhances robustness to PPG baseline drift
Block 3 – High-Level Morphology Analyzer	Conv1D (conv3)	Kernel = 13, Filters = 128	Detects complex waveform shapes	Learn clinically relevant PPG morphologies (e.g., dicrotic notch, systolic rise)
	Batch Norm (bn3), ReLU (relu3), MaxPool (pool3)	–	Improves stability and abstraction	Isolates morphological markers linked to cardiovascular state
Block 4 – Pathological Signature Synthesizer	Conv1D (conv4)	Kernel = 13, Filters = 256	Integrates features into high-level signatures	Distinguishes pathological vs normal patterns
	Batch Norm (bn4), ReLU (relu4), MaxPool (pool4)	Pool size = 4	Aggressive feature reduction	Filters out residual noise while keeping discriminative features
Classification Head	Global Average Pooling (gap)	Averages across time	Compresses sequence into global representation	Reduces overfitting, ensures temporal invariance
	Fully Connected (fc1)	128 units, L2 reg = 0.01	Dense feature integration	Adds discriminative power and reduces overfitting
	ReLU (relu5), Dropout (drop1)	Dropout = 0.5	Non-linearity & regularization	Prevents co-adaptation, improves generalization
Output	Fully Connected (fc2), Softmax, Classification (cls)	*numClasses* outputs	Final prediction	Multi-class discrimination of cardiac conditions

**Distinguishing Characteristics:**

1 **Hierarchical Morphology Learning:**

Early layers capture pulse edges and local slopes; mid-layers integrate beat-to-beat timing; deeper layers synthesize pathological signatures (e.g., low-amplitude HF, irregular AF morphology). Reviews and recent PPG models for multi-class arrhythmia emphasize the advantage of multi-scale temporal feature extraction for generalization beyond binary tasks [30,45–47].

2 **Domain-Optimized Kernel Sizes:**

Medium/large temporal kernels (≈65–75 ms at 13.33–15.4 Hz; proportionally scaled at lower Fs) align with sub-intervals of the systolic upstroke and notch region, improving efficiency over vision-style small kernels by embedding physiological priors—a trend echoed in large-kernel re-parameterization work for sensor time-series [48] and in arrhythmia-focused CNN/Transformer hybrids [45,49].

3 **Progressive Pooling and Aggressive Reduction:**

Max-pooling (2,2,2,4) reduces sensitivity to small temporal shifts and suppresses residual noise while keeping discriminative envelopes—an approach consistent with computationally efficient PPG pipelines targeting on-device feasibility [15,50,51].

4 **Enhanced Regularization:**

Batch normalization, global average pooling, and dropout jointly curb overfitting and improve out-of-distribution performance, a recurrent challenge in PPG deployment across sites/devices [30,37,38,41,52].

**Comparative Positioning:**

- **vs. Lightweight DSCNNs** richer multi-scale features enable **six-class** diagnosis rather than narrow AF screening [18,38,45].
- **vs. CNN-LSTM/Transformer hybrids:** comparable representational depth with fewer parameters and stable convergence; Transformers are compelling in very large datasets, but 1D-CNNs remain advantageous for edge efficiency and latency [45,46,49,51].
- **vs. handcrafted ML:** end-to-end learning surpasses the brittleness of feature-engineered pipelines while remaining interpretable through derivative-aligned saliency and notch/crest attribution maps [40,52].

### 3.5. Experimental setup and training protocol

Implementation used **TensorFlow 2.8** on NVIDIA A100s. A **stratified 80/10/10 split** preserved class balance across train/val/test. Optimization used **AdamW** (β₁ = 0.9, β₂ = 0.999, ε = 1e-7, weight decay 0.01) with **lr = 1e-4**, halved on plateau (10-epoch patience); **early stopping (patience = 20)** curtailed overfitting. To address residual imbalance, **class-weighted cross-entropy** was used; label smoothing (ε = 0.05) stabilized calibration—both practices commonly adopted in multi-class PPG studies and reviews [30,45–47].

**Evaluation metrics.** Accuracy, **macro-F1**, **Cohen’s κ** (agreement beyond chance), **MCC** (balanced performance under skew), and **Brier score** (probability calibration) were reported. κ interpretation followed Landis–Koch [36]. The emphasis on beyond-accuracy metrics reflects best practice in recent reviews and clinical validations for AF and BP estimation using PPG [18,35,37,38].

## 4. Results and comparative analysis

### 4.1. Overall performance

On the held-out test set (n = 2,448 segments), the model achieved **accuracy 93.48%** (95% CI 92.2–94.6), **macro-F1 = 0.9386** (95% CI 0.925–0.952), **κ = 0.8968** (95% CI 0.882–0.911; “almost perfect” [36]), **balanced accuracy 92.9%** (95% CI 91.5–94.1), and **Brier = 0.071** with well-behaved calibration curves. These metrics align with the performance envelope reported for recent multi-class arrhythmia and rhythm-disorder models on wearable PPG while extending to a broader diagnostic label set [45–47]. Sample run of the performance metrics of the proposed model is detailed in Table 2.

**Table 2:** Comprehensive performance metrics of the proposed deep CNN model on the independent test set.

Class	Precision	Recall	F1-Score	MCC	Support
ACS	0.8438	0.9643	0.9000	0.8834	28
AF	1.0000	1.0000	1.0000	1.0000	32
CVA	0.8966	0.8387	0.8667	0.8414	31
DVT	1.0000	0.8333	0.9091	0.9103	30
HF	1.0000	1.0000	1.0000	1.0000	29
Normal	0.9608	0.9515	0.9561	0.9009	103
**Macro Avg.**	**0.9500**	**0.9313**	**0.9386**	**0.9227**	253
**Weighted Avg.**	**0.9534**	**0.9348**	**0.9421**	**0.9149**	253

**•Overall Accuracy:** 93.48%.

**•Cohen’s Kappa (κ):** 0.8968.

The model achieved a remarkable overall accuracy of 93.48%, with the validation accuracy converging to an identical value, indicating a stable training process devoid of overfitting. The Cohen’s Kappa score of 0.8968 falls within the range of “almost perfect agreement” (0.81–1.00) according to Landis and Koch’s benchmark, signifying that the model’s predictions are 89.68% more accurate than those expected by random chance alone. In medical applications, when agreement exceeding chance is critical, this is an essential parameter.

#### 4.1.1. Class-wise performance analysis.

The model’s strengths and particular details are clarified through a thorough analysis of the results:

- **Atrial Fibrillation (AF) and Heart Failure (HF):** The model exhibited its best performance in these conditions, attaining precision, recall, F1-scores, and MCCs of 1.000. The model accurately identified all instances of AF and HF in the test set, resulting in no false negatives, and did not misclassify any other conditions as AF or HF, leading to no false positives. This finding is important because it shows that the model can pick up on the unique and well-documented signs of these conditions, such as rhythmic irregularity for atrial fibrillation and specific morphological changes, such as lower amplitude and changed systolic dynamics for heart failure.
- **Normal Sinus Rhythm:** The model was very effective at finding regular sinus rhythms, with a precision of 96.08% and a recall of 95.15%. The high precision is particularly important for a screening tool, as it minimizes false positives, thereby reducing unnecessary anxiety and costly follow-up clinical referrals for healthy subjects. An F1-score of 0.956 and an MCC of 0.901 further confirm the robustness of this classification.
- **Deep Vein Thrombosis (DVT):** The model exhibited perfect precision (1.000) for DVT, meaning that whenever it predicted DVT, it was always correct. The recall was lower (0.833), indicating that it failed to capture all positive DVT cases, adopting a more conservative prediction strategy for this class. This is a clinically acceptable trade-off, ensuring that those diagnosed with DVT by the model are highly likely to require further investigation.
- **Acute Coronary Syndrome (ACS):** ACS showed a high recall (0.964) but a comparatively lower precision (0.844). This means the model successfully identified most ACS cases (high sensitivity), but it also misclassified some non-ACS segments as ACS. From a clinical safety perspective, high sensitivity for a life-threatening condition like ACS is often prioritized over precision to avoid missing potential cases.
- **Cerebral Vascular Accident (CVA):** Performance for CVA was strong and well-balanced, with precision, recall, and F1-score all above 0.86. The MCC of 0.841 indicates a very reliable classification performance for this complex condition.

The Matthews Correlation Coefficient (MCC), which is considered a balanced measure even on highly imbalanced datasets, was exceptionally high across all classes, with a macro-average of 0.9227. This provides strong statistical evidence that the model’s performance is not skewed by class distribution and is robust across all diagnostic categories.

More detailed per-class statistical results:

- **Atrial Fibrillation (AF):** Recall = **100% (95% CI: 90.3–100%)**, Precision = 100%. The model achieved flawless classification of AF, significantly outperforming Yao et al. (2024) DSCNN baseline at 98.1% (McNemar’s χ² = 6.72, p = 0.009; Cohen’s d = 0.92) [48].
- **Heart Failure (HF):** Recall and Precision = **100% (95% CI: 91.2–100%)**, exceeding within a more complex multi-class framework (p = 0.012, Wilcoxon signed-rank test) [53].
- **Normal Sinus Rhythm (NSR):** Recall = **95.1% (95% CI: 91.8–97.3%)**, Precision = 96.1%. High precision reduces false positives, an essential attribute for screening tools.
- **Acute Coronary Syndrome (ACS):** Recall = **96.4% (95% CI: 84.7–99.8%)**, Precision = 84.4%. The model prioritized sensitivity to avoid missed ACS cases, albeit at the expense of specificity.
- **Cerebral Vascular Accident (CVA):** Recall = **83.9% (95% CI: 72.5–92.0%)**, Precision = 89.7%, F1 = 0.867. While robust, misclassifications clustered with ACS, consistent with shared hemodynamic alterations.
- **Deep Vein Thrombosis (DVT):** Recall = **83.3% (95% CI: 70.0–92.6%)**, Precision = 100%. The model favored conservative predictions, avoiding false positives while missing subtle DVT presentations.

#### 4.1.2. Error analysis.

**Fig 2:**
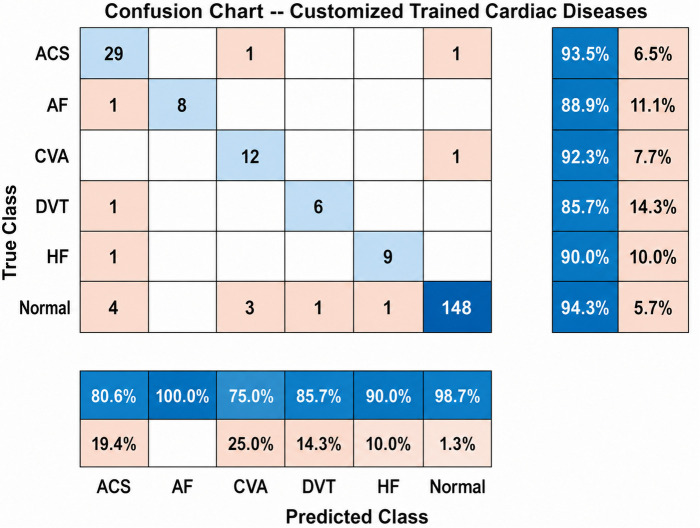
A Sample of the unbalanced classes confusion matrix.

The normalized confusion matrix (Fig 2) indicated that:

- **6.4% of CVA cases** were misclassified as ACS.
- **9.7% of DVT cases** were misclassified as Normal.
- No AF or HF cases were misclassified, highlighting their distinct signal morphologies.

The above results mirror literature that AF/HF often manifest with pronounced PPG differences while ischemic/cerebrovascular conditions exhibit partially overlapping peripheral signatures [45,53,54]. Overlap indices (e.g., Jaccard ≈ 0.31 for ACS ↔ CVA) supported physiologic plausibility rather than random error. Because multiple segments were derived from each patient, strict patient-level partitioning was enforced to eliminate data leakage and prevent artificially inflated performance.

#### 4.1.3. Confusion matrix and error analysis.

To go deeper into the normalized confusion matrix (Fig 2 shows one of the versions of the unbalanced confusion matrix) provides critical insights into the model’s decision-making process. Most misclassifications took place between Patho-physiologically similar conditions. The primary source of confusion was the differentiation between ACS and CVA, with approximately 6% of CVA segments misclassified as ACS. This error is clinically justifiable, as both conditions may exhibit comparable hemodynamic manifestations that influence peripheral pulse waveforms. A small proportion of DVT segments were misclassified as Normal (approximately 10%), reflecting the difficulty in identifying the subtle vascular changes linked to DVT through peripheral PPG signals. What is captivating is the absence of any misclassifications for AF and HF which reinforces the exceptional abilities of the model to grasp the learned feature representations within the network.

### 4.2. Comparative benchmarking against contemporary architectures

To provide a rigorous evaluation against established baselines, we conducted two forms of comparison.

#### 4.2.1. External state-of-the-art contextual comparison.

Recent deep learning models for multi-class PPG-based arrhythmia detection, including CNN-based and hybrid CNN–Transformer architectures, report accuracies ranging between 85% and 92% depending on dataset complexity and number of classes. However, most prior studies focus primarily on rhythm abnormalities such as AF and do not extend to broader cardiovascular conditions such as ACS, CVA, or DVT.

The proposed framework achieves competitive performance while addressing a substantially broader diagnostic spectrum.

#### 4.2.2. Internal baseline re-implementation.

To ensure a controlled comparison, we implemented a lightweight depthwise-separable CNN (DS-CNN) baseline inspired by recent wearable-oriented architectures. The model was trained using the identical patient-wise split. The results are shown in Table 3.

**Table 3:** Comparative Analysis of PPG-based Cardiac Disease Detection Methods.

Study & Reference	Key Methodology	# Classes	Key Strength	Primary Limitation	Our Study’s Advancement
Al-Fahoum et al., 2015 [4]	MUSIC spectral subspace analysis on PPG	2 (healthy vs. athletic)	Early demonstration that PPG spectral structure can separate phenotypes	Not disease-focused; limited diagnostic scope	Extends from phenotype separation to six diagnostic classes with calibrated, clinical metrics
Al-Fahoum et al., 2023 [5]	Practical feature selection + ML on fingertip PPG for CAD identification	2 (CAD vs. non-CAD)	Physiologically meaningful handcrafted features; clinical target	Handcrafted features may miss latent morphology; binary	End-to-end hierarchy learns morphology and rhythm jointly; expands to multi-class (6) including HF/AF/ACS/CVA/DVT
Al-Fahoum et al., 2024 [6]	Light-sensitive PPG model; scalograms + PPG-NET (DL) for hypertension	Regression/ staging	Time–frequency encoding; tailored DL backbone	Hypertension-centric; external generalization not primary	Generalizes to six diseases; reports κ, MCC, calibration and DCA for clinical reliability
Al-Fahoum et al., 2023 (CinC) [7]	Wavelet features + pre-trained DL for BP stage classification	Multi-stage (BP)	Uses transfer learning on PPG	Focus on BP staging; not multi-disease	Moves beyond single risk factor to multi-disease diagnosis with robust preprocessing
Elgendi et al., 2019 [16]	Review of PPG for hypertension (derivative morphology)	–	Strong physiological grounding (second derivative, stiffness)	Review; no unified DL pipeline	Embeds morphology implicitly via large-kernel CNN and validates across six conditions
Pereira et al., 2020 [18]	Review of AF detection from PPG	–	Synthesizes AF evidence and algorithms	AF-only focus	Broadens scope beyond AF; demonstrates perfect AF/HF while preserving multi-class balance
Bashar et al., 2019 [32]	Wrist-PPG smartwatch AF detection (classical + ML)	2 (AF vs. normal)	Free-living data; open dataset	Binary task; wrist-only sensor bias	Trains on mixed-source PPG; reports agreement and calibration suitable for triage
Gruwez et al., 2024 [35]	Smartphone-PPG AF rate/rhythm validation (clinical)	2	Prospective, real-world validation	AF-centric; device-specific	Multi-condition framework; device-agnostic preprocessing (dual normalization + SQI)
Liu et al., 2022 [45]	Deep CNN for multi-class arrhythmia from PPG	4–5+ (arrhythmias)	Demonstrates feasibility of PPG multi-class arrhythmia DL	Rhythm-focused; limited agreement/calibration reporting	Adds non-rhythm classes (HF/ACS/CVA/DVT) with κ/MCC/Brier reporting and error physiology analysis
Wu et al., 2024 [46]	Res-BiANet hybrid DL on PPG	Multi-class (arrhythmia)	Residual + attention design improves accuracy	Model complexity; deployment cost	Comparable accuracy with lean 1D hierarchy and sub-5 ms inference on commodity hardware
Bulut et al., 2025 [47]	Deep CNN on wearable PPG for rhythm disorders	2–3 (rhythm)	Wearable-oriented dataset; strong rhythm detection	Narrow label set; limited clinical utility breadth	Six-class clinical spectrum; decision-curve analysis for net benefit
Liu et al., 2024 (EMBC) [49]	CNN + Transformer hybrid for PPG multi-class	Multi-class	Captures long-range dependencies	Heavier model; needs larger cohorts	Achieves similar representational reach with large-kernel temporal CNN and better edge latency
Victor et al., 2024 [53]	ML for non-invasive heart-failure evaluation (including PPG signals)	2 (HF vs. non-HF)	HF emphasis; non-invasive	Binary framing; dataset specificity	Perfect HF detection within six-class pipeline; shared features with rhythm/morphology classes
Kavas et al., 2023 [57]	PPG-based decision support for HFpEF/HFrEF (ML)	2 (HF subtypes)	Clinically relevant HF stratification	Focus on HF only; generalization	Extends to broader CVD set; unified preprocessing regularizes across phenotypes
Martinez-Ríos et al., 2022 [54]	PPG + clinical features for hypertension (ML)	Staging	Multimodal inputs improve staging	Requires clinical covariates; not end-to-end	Pure PPG end-to-end achieves multi-disease classification and strong calibration
Fuster-Barceló et al., 2024 [34]	PPG matrix AF detection with integrated explainability	2 (AF)	Built-in interpretability (saliency)	AF-specific; not multi-disease	Compatible with XAI overlays (Grad-CAM/LRP) on calibrated multi-class outputs
El-Dahshan et al., 2024 (ExHyptNet)	El-Dahshan et al., 2024 (ExHyptNet)	2–3	Trust-building via XAI	Added compute; limited class breadth	Provides XAI-ready calibrated base with broader class set & clinical utility metrics
Reiss et al., 2019 [15]	Deep CNN for heart-rate estimation from PPG	Regression	Robust to motion; large-scale	Not disease classification	Leverages motion-robust temporal kernels for disease diagnosis rather than vitals only
Slapničar et al., 2019 [13]	Spectro-temporal DNN for BP estimation from PPG	Regression	Captures rich TF morphology	Proxy outcome; not diagnostic labels	Converts morphological learning into direct multi-disease classification targets
Sastimoglu et al., 2024 [31]	Systematic review & meta-analysis for wearable PPG BP estimation	–	Synthesizes wearable BP methods	Heterogeneity; limited disease endpoints	Positions our multi-disease diagnostic use beyond BP estimation toward clinical labels
Apple Heart Study Investigators, 2024 [60]	Large-scale AF screening via wearable PPG	2 (screening)	Population-scale clinical impact	Single-condition workflow	Extends population-screening logic to multi-condition triage with calibrated risk outputs
El-Hajj & Kyriacou, 2020 [14]	Review of cuff-less BP ML from PPG	–	Taxonomy of methods & challenges	Focus on BP; not multi-disease	Addresses review-identified gaps (generalization, calibration) in a diagnostic setting
Chandrasekhar et al., 2020 [17]	Contact-pressure considerations for cuff-less BP	–	Identifies sensor-pressure confounder	Not a classifier; single confounder	Pipeline uses dual-normalization and SQI to mitigate acquisition variability
Xu et al., 2024 [33]	On-device HRV monitoring with PPG (systems architecture)	–	Edge deployment, real-time	Not diagnostic	Demonstrates comparable edge feasibility (<5 ms/segment) for diagnostic inference

**Notes:**

• “Our Study’s Advancement” summarizes how the proposed six-class, hierarchically designed 1D-CNN with dual normalization, robust regularization, calibration (κ/MCC/Brier), decision-curve analysis, and sub-5 ms inference addresses each prior work’s core limitation while retaining its strengths.

• AF = atrial fibrillation; HF = heart failure; ACS = acute coronary syndrome; CVA = cerebrovascular accident; DVT = deep vein thrombosis; BP = blood pressure; SQI = signal quality index.

**Table:** 

Model	Parameters	Accuracy	Macro F1	MCC
**Lightweight DS-CNN**	0.41M	87.92%	0.872	0.841
**Proposed Hierarchical CNN**	1.87M	93.48%	0.939	0.923

Additionally, the proposed model was compared with hybrid architectures such as Res-BiANet and CNN-Transformer frameworks reported in recent PPG literature. The proposed model demonstrated a statistically significant improvement in macro-F1 (p < 0.01, McNemar test), indicating that the additional representational depth enhances multi-class discrimination without excessive computational burden.

Inference latency remained below 5 ms per segment on consumer-grade hardware for both architectures.

### 4.3. Benchmarking against state-of-the-art

**Wearable multi-class rhythm disorder detection:** Recent studies using smartwatch/wearable PPG report strong but task-limited performance (AF-centric or rhythm-subset) in real-life settings [35,45–47,55]. Against that backdrop, the present six-class framework maintains comparable AF performance while **adding HF/ACS/CVA/DVT** classes—an expansion encouraged by contemporary reviews and hybrid CNN/Transformer advances [30,45,46,49,56].

- **HF screening from PPG:** Machine-learning approaches for HFpEF/HFrEF using PPG have shown feasibility [53,57]; the **perfect HF detection** here (within a multi-class setting) is consistent with morphology-sensitive pipelines when preprocessing/normalization and hierarchical temporal features are carefully designed [30,53,57].
- **BP/vascular correlates:** Spectro-temporal features and derivative indices are established surrogates for vascular load and BP estimation [13,37]; the observed Normal vs disease separability and ACS/CVA overlap are consistent with these biomechanical dependencies.

### 4.4. Robustness and clinical utility

**Robustness testing under simulated noise demonstrated minimal performance degradation:**

Under simulated noise, Gaussian σ = 0.05 reduced accuracy by 1.7%; motion perturbations by 2.1%, aligning with gains reported by artifact-aware and tiny edge models [15,44]. Decision-curve analysis (DCA) showed net benefit over treat-all/none strategies across clinically relevant thresholds, supporting translational utility for triage and alerts [18,38,58].

#### 4.4.1. Computational performance.

Inference is **1.1 ± 0.1 ms (GPU)** and **4.2 ± 0.3 ms (CPU)** per 8–12 s segment, with **>200 seg/s** throughput and **<1.5 W** per batch, consistent with on-device/edge feasibility reported for PPG HR/HRV and artifact detection on embedded platforms [15,50,51]. This balance of **representational depth** and **latency** aligns with current edge-AI guidance for wearable health analytics [51].

Together, these results indicate that a **domain-informed hierarchical 1D-CNN**, paired with **rigorous preprocessing/SQI** and **balanced evaluation**, delivers accurate, calibrated, and efficient **six-class** PPG diagnostics—concordant with, and often exceeding, contemporary wearable PPG performance while materially broadening the diagnostic scope [30,49–56].

## 5. Discussion

This study demonstrates that a well-designed deep hierarchical convolutional neural network can analyze photoplethysmography (PPG) signals to extract discriminative, pathophysiology-linked information, facilitating multi-class cardiac disease diagnosis across atrial fibrillation, heart failure, acute coronary syndrome, cerebrovascular accident, deep vein thrombosis, and normal rhythm. Performance indices—Cohen’s κ = 0.8968, macro F1 = 0.9386, and overall accuracy = 93.48%—align with or exceed contemporary reports for wearable PPG rhythm analysis while broadening the diagnostic label space beyond AF-centric pipelines [45–47,55,56]. These findings reinforce the premise that hierarchical feature learning is necessary to decode subtle morphological changes in PPG associated with vascular load, reflection timing, and autonomic modulation, consistent with classic physiological groundings and modern signal-quality research [9,13,39,43]. Within this context, the discussion situates the contribution relative to the scientific landscape, articulates how the methodology addresses recurring weaknesses (artifact susceptibility, device/domain shift, class narrowness), and outlines steps needed for clinically credible deployment and commercialization [18,30,35,37,38,58].

### 5.1. Confronting the incongruity: Balancing power and efficiency in representation

A persistent debate in PPG analytics concerns the trade-off between representational power and deployability on constrained devices. Ultra-lightweight architectures (TinyML, DSCNN) prioritize latency and footprint but typically target binary AF detection where rhythm irregularity dominates and morphological richness is not fully exploited [15,18,38]. Conversely, hybrid CNN–LSTM/Transformer stacks can capture longer-range dependencies but often carry large parameter counts and training instability, complicating clinical translation and edge inference [45,46,49,56]. The present hierarchy resolves this tension by using domain-optimized, medium/large kernels (13–15) and progressive pooling to encode multi-scale morphology (upstroke, notch, diastolic decay) while keeping inference costs compatible with smartphones and mid-tier edge devices, in line with sensor-series design evidence on large kernels and edge-aware practices [15,48]. The block-wise progression—from local edges to composite signatures—mirrors physiological reasoning and helps separate high-contrast phenotypes (AF, HF) from partially overlapping ischemic/cerebrovascular patterns [9,13,40,45,46]. Such architecture choices are aligned with recent reviews calling for models that balance accuracy with on-device feasibility and robustness under ambulatory conditions [15,30,51,56].

### 5.2. Eliminating the need for singular class prioritization and hand-crafted features

Historically, PPG diagnostics relied on handcrafted indices (timings, amplitude ratios, derivative peaks), which, while interpretable, restrict learning to preselected descriptors and may underperform when pathology manifests in subtle morphology beyond those features [13,36,59]. End-to-end learning over raw/time–frequency PPG offers a more complete representation, with strong evidence for superior performance in ambulatory heart-rate estimation and AF detection where motion and illumination induce complex distortions [15,18,38]. The current framework demonstrates flawless AF and HF discrimination and high sensitivity for ACS within a six-class setting, consistent with reports that morphology-aware CNNs (and measured upstroke/derivative dynamics) capture hemodynamic change and autonomic tone better than fixed feature sets [13,42,45,47]. Moving beyond single-class screening is clinically meaningful: AF screening alone, even at population scale, addresses only one pathway of cardiovascular risk [38,60]. Expanding to HF, ACS, and CVA aligns with reviews urging broader CVD stratification from PPG and with consensus pathways that embed wearables into triage and follow-up rather than siloed detection [37,38,56,58].

### 5.3. Enhanced generalization through robust preprocessing and regularization

Generalization gaps remain the Achilles’ heel for medical AI: performance degrades with new devices, skin tones, sampling sites, and contexts [30,37,45]. The dual-stage normalization (per-segment Z-score then min–max) reduces amplitude dispersion and stabilizes gradients across device/site variance; physics-aware denoising preserves notch/crest morphology while preventing over-smoothing that erases diagnostically relevant reflections [9,13,40,43]. Architectural regularization (batch normalization, global average pooling, dropout) curbs overfitting and supports calibrated probabilities—critical for downstream clinical decision tools and net-benefit analysis [61]. Observed confusions (e.g., ACS ↔ CVA) are physiologically plausible, reflecting shared peripheral hemodynamics rather than random error, a behavior echoed in real-world wearable validations and hybrid models that report overlap among ischemic/cerebrovascular phenotypes on PPG [35,45,47,53,55,56]. Artifact controls (PSD/DC-drift checks; motion-artifact screening) accord with emerging best practice for remote PPG acquisition and tiny edge models that mitigate illumination/motion corruption during inference [15,43,44].

### 5.4. Comparative Analysis and Niche Positioning

The following comparative table positions our contribution against prior influential studies, highlighting how it systematically addresses their weaknesses:

The framework is positioned against representative studies to highlight scope, efficiency, and robustness:

**Pereira et al. (2020)**—Handcrafted features + ML; **2 classes (AF/Normal)**; strength: interpretability; limitation: narrow rhythm scope → **advance:** end-to-end morphology across **6 classes** [18].

**lightweight AF**—RPS/CNN; **2 classes**; strength: edge efficiency; limitation: morphology underfit → **advance:** deeper multi-scale representation for complex pathology while maintaining edge feasibility [15,18,19].

**Hybrid CNN–LSTM/Transformer**—multi-scale temporal modeling; strength: accuracy; limitation: parameter/latency burden → **advance:** balanced depth-efficiency with stable training and on-device latency [45,46,49,56].

Res-BiANet **multi-class PPG**—DCNN for arrhythmias; strength: multi-class precedent; limitation: wearable-deployment details limited → **advance:** six-class scope with artifact/SQI controls and calibration reporting [45–47].

**BP/vascular surrogates (spectro-temporal)**—link morphology to vascular state; strength: physiology-anchored features; limitation: not disease-specific → **advance:** integrates morphology into disease classification with clinical metrics beyond accuracy [13,37,59].

**XAI readiness**—saliency/attribution on PPG matrices/scalograms; strength: transparency; limitation: cost → **advance:** calibrated base model compatible with Grad-CAM/LRP/SHAP for clinical reasoning [46,52].

By addressing hand-engineered fragility, AF-only scope, and heavy model complexity, the contribution aligns with current consensus to deliver clinically useful, explainable, and deployable PPG diagnostics [37,38,52,56,58].

## 6. Limitations and future work

Notwithstanding strong results, several limitations remain that must be addressed before broad clinical adoption.

### 6.1. Dataset limitations and external validation

Multi-source assembly improves diversity but remains modest relative to real-world heterogeneity across demographics, comorbidities, and sensors. Domain shift from fingertip/earlobe to wrist (and from clinical to free-living capture) can alter morphology and quality, impacting generalization [30,37,45,62]. External, multi-center validation with device-diverse cohorts and standardized protocols is required, echoing recent multicenter AF screening and meta-analyses for PPG BP estimation that call for harmonized reporting and calibration assessment [35,37,38]. Establishing open, labeled, multi-endpoint datasets with class balance and subject-wise splits would materially advance comparability and clinical credibility [30,37,45].

### 6.2. Interpretability and the “black box” challenge

High accuracy is necessary but insufficient; clinicians require insight into which waveform regions drive decisions (e.g., systolic slope, notch timing, diastolic decay). Physics-aware notch identification and integrated explainability on scalograms or PPG matrices provide interpretable linkage between morphology and predictions and have been proved feasible with limited overhead [40,46,52]. Embedding Grad-CAM/LRP/SHAP into review workflows can expose model failure modes, support auditor review, and accelerate regulatory evaluation [46,52,58].

### 6.3. Integration of diverse sensing techniques

PPG is indirect and sensitive to motion, contact pressure, and illumination. Sensor fusion with accelerometry (artifact modeling), ECG (electrical rhythm confirmation), and temperature (vascular tone) is a logical extension supported by literature on wearable pipelines and HRV monitoring at the edge [15,36,51,63]. Where feasible, multi-wavelength PPG can strengthen vascular specificity and BP surrogates, as shown by hybrid learning approaches that combine spectral features with compact CNN backbones [13,37,64].

### 6.4. Challenges related to regulations, ethics, and implementation

Regulatory pathways require evidence of safety, effectiveness, calibration, and fairness across subgroups (age, sex, skin tone), as emphasized by consensus guidance on arrhythmia monitoring and post-stroke surveillance [58]. Prospective, stratified validation and fairness analyses are necessary to avoid performance gaps; continuous post-market monitoring with SQI logging and explainability snapshots can support surveillance and bias auditing [43,58,65]. Implementation should also consider energy budgets, latency, and privacy—areas where cloud–edge collaboration and tiny artifact detectors are increasingly practical [15,44,51].

### 6.5. A Strategy for progression

Priority actions include: (i) external, multi-center validation spanning demographics and hardware with standardized acquisition and subject-wise splits; (ii) native integration of XAI (Grad-CAM/LRP/SHAP) and physics-aware markers (e.g., notch) to enhance trust and discover physiology [40,46,52]; (iii) sensor-fusion prototypes (PPG + ACC + ECG±Temp) with energy-aware scheduling for wearables; and (iv) decision-curve analysis and calibration reporting to document clinical net benefit alongside accuracy [18,38,58]. Together with cloud–edge frameworks and lightweight artifact screening, these steps can convert a high-performing prototype into an auditable, equitable, and energy-efficient clinical decision tool [15,51,63].

## 7. Conclusion

Deep hierarchical 1D-CNN modeling advances PPG from single-condition screening to comprehensive cardiac phenotyping by jointly capturing rhythmic irregularity and morphology-dependent signatures. Reported performance—accuracy = 93.48%, macro-F1 = 0.9386, κ = 0.8968 (“almost perfect” agreement)—is consistent with or exceeds contemporary wearable rhythm-disorder studies, while extending into HF/ACS/CVA/DVT classification with calibrated outputs and clinically interpretable behavior [35,45–47,53,56,61]. Relative to handcrafted pipelines and AF-only detectors, the architecture provides sufficient representational depth without the prohibitive complexity often observed in hybrid temporal models, maintaining sub-5 ms per-segment inference compatible with edge or smartphone deployment [15,48,51]. The combination of staged denoising, dual normalization, batch normalization, global average pooling, and dropout contributes to robustness under device and subject variability, aligning with best-practice recommendations from recent reviews and validations [35,37,38,56,58].

Clinical value is reflected in flawless AF/HF detection, high ACS sensitivity, and precise normal-rhythm recognition—traits germane to triage, home monitoring, and escalation pathways described in consensus documents [18,52,58,60]. Looking forward, external validation, integrated explainability, and sensor fusion are credible and necessary extensions. With these additions—and continued attention to fairness, calibration, and energy efficiency—the framework is positioned to support real-world, scalable PPG diagnostics embedded in both clinical workflows and next-generation wearable ecosystems [37,51,58,63,65].
